# Enhanced hydrogen evolution reaction on hybrids of cobalt phosphide and molybdenum phosphide

**DOI:** 10.1098/rsos.161016

**Published:** 2017-03-01

**Authors:** Si-Ling Fang, Tsu-Chin Chou, Satyanarayana Samireddi, Kuei-Hsien Chen, Li-Chyong Chen, Wei-Fu Chen

**Affiliations:** 1 Center for Condensed Matter Sciences, National Taiwan University, Taipei 10617, Taiwan, Republic of China; 2 Institute of Atomic and Molecular Sciences, Academia Sinica, Taipei 10617, Taiwan, Republic of China; 3 Department of Chemistry, National Tsing Hua University, Hsinchu 30012, Taiwan, Republic of China

**Keywords:** hydrogen evolution reaction, electrocatalyst, ternary, cobalt phosphide, molybdenum phosphide

## Abstract

Production of hydrogen from water electrolysis has stimulated the search of sustainable electrocatalysts as possible alternatives. Recently, cobalt phosphide (CoP) and molybdenum phosphide (MoP) received great attention owing to their superior catalytic activity and stability towards the hydrogen evolution reaction (HER) which rivals platinum catalysts. In this study, we synthesize and study a series of catalysts based on hybrids of CoP and MoP with different Co/Mo ratio. The HER activity shows a volcano shape and reaches a maximum for Co/Mo = 1. Tafel analysis indicates a change in the dominating step of Volmer–Hyrovský mechanism. Interestingly, X-ray diffraction patterns confirmed a major ternary interstitial hexagonal CoMoP_2_ crystal phase is formed which enhances the electrochemical activity.

## 1. Introduction

Generation of hydrogen fuel from water as alternative to fossil oils without releasing carbonaceous gases, such as carbon monoxide and carbon dioxide [1], has been considered as a promising green technology. Electrolytic hydrogen evolution reaction (HER) has received great attention, because it can be activated from renewable sources of energy like wind and solar [2,3]. However, water electrolysers are hampered by high costs and limited abundance of electrode materials owing to the use of noble metals like platinum. This makes water electrolysers unfavourable to compete with natural gas reforming. It is of high priority to research earth-abundant catalysts as possible alternatives to noble metals [4–6]. Unfortunately, transition metal-based electrocatalysts have suffered from high overpotentials and corrosion problems in acidic media [7].

Transition metal-based pnictides [8,9] (nitrogen-group elements) are potential electrocatalysts for the HER, as they possess excellent corrosion resistance in the HER condition and good electrical conductivity as an electrode. Very recently, metal phosphides have received great attention owing to their superior catalytic activity towards the HER which rivals platinum catalysts [10–12]. It has previously been reported by Schaak and co-workers [13–15] that orthorhombic CoP and Co_2_P nanocrystallines produced cathodic current density of 20 mA cm^−2^ at overpotentials ranging from 85 to 117 mV and stable over 24 h of operation. Bulk hexagonal molybdenum phosphide (MoP) has shown stable HER activity in acidic solution with overpotentials of 140–246 mV for driving a current density of 10 mA cm^−2^ [16,17]. Even lower overpotentials were reported on amorphous or nanosized MoP HER when compared with bulk MoP [18,19]. It is established that doping Co to MoP increases the intrinsic activity of MoP (so-called Co-promoted MoP); however, the positioning of Co and the exact structural polytype are still not resolved [20]. A decent comprehension of the correspondence between the HER activity and the crystal structure of these materials is not yet thoroughly developed. For bimetal alloys, material design methodologies for optimizing the electronic structure and electrochemical activity are well investigated [21–25]; however, only a few studies were focused on the modification of structure of mixed metal pnictides to accelerate the electrolytic reaction. Cao *et al*. [26] reported a layered Co_0.6_Mo_1.4_N_2_ allows the Co to tune the electronic states of molybdenum at the catalyst surface without disrupting the catalytic activity. Staszak-Jirkovský *et al*. [27] demonstrated Co*^n^*^+^ cations in a CoMoS*_x_* chalcogel structure helped accelerate the rate-determining Volmer step. Ternary Chevrel-phase NiMo_3_S_4_ was reported by Jiang *et al*. [28] that the interconnected [Mo_6_S_8_]_2_ cluster units allow faster charge transfer.

Herein, we show that this research improves upon previous Co-promoted MoP catalysts towards the HER by optimizing the CoP and MoP compositions. We have used a solid-state synthesis route to prepare hybrids of CoP and MoP. Structural studies indicate that at the ratio of Co/Mo = 1, a new crystalline hexagonal CoMoP_2_ phase is formed in addition to CoP and MoP phases. The hybrid with Co/Mo = 1 exhibits good HER performance and shows promise as an efficient cost-effective cathode material for water splitting.

## 2. Material and methods

### 2.1. Synthesis of hybrids of CoP and MoP

Hybrids of cobalt phosphide and molybdenum phosphide were prepared by a two-step thermal treatment of mixtures of cobalt nitrate (Co(NO_3_)_2_.6H_2_O, Acros), ammonium molybdate ((NH_4_)_6_Mo_7_O_24_.4H_2_O, Aldrich) and ammonium dihydrogen phosphate (NH_4_H_2_PO_4_, Aldrich). A typical procedure is shown as follows. For preparing Co_0.5_Mo_0.5_P catalyst, 10 µmol ammonium molybdate, 70 µmol cobalt nitrate and 140 µmol ammonium dihydrogen phosphate were mixed in 50 ml water and were ultrasonicated until all salts were dissolved. The ratio of cobalt nitrate to ammonium molybdate was varied to create molar ratios [Co]/[Mo] of 0.1/0.9, 0.3/0/7, 0.5/0.5, 0.7/0.3 and 0.9/0.1. After mixing, the transparent solution was dried at 80°C in an oven. The solid mixture was then annealed in a quartz tube furnace with a 100 sccm Ar flow from ambient to 350°C at a rate of 10°C min^−1^ and then held at 300°C for 1 h. Then the gas flow was switched to H_2_/Ar mix flow (H_2_ 50 sccm; Ar 50 sccm). The temperature was increased to 800°C at a ramping rate of 15°C min^−1^ and held at 800°C for 2 h. Bulk CoP and MoP were prepared by the same procedure using corresponding precursors with [Co or Mo]/[P] = 1. To study the effect of annealing temperature, the precursors of Co^0.5^Mo^0.5^P were treated at 650°C and 1000°C under H_2_/Ar mix flow for 2 h after the annealing at 350°C under Ar.

### 2.2. Structural characterization

The micromorphology of hybrid catalysts were observed on a field emission scanning electron microscope (JOEL JSM−6700F) and on a JEOL JEM-2100 transmission electron microscope. The crystalline compositions of the Co^φ^Mo^τ^P hybrids were verified by X-ray diffraction (XRD) patterns with a Bruker D2 PHASER diffractometer. X-ray photoelectron spectra (XPS) were collected on a VG Scientific ESCALAB 250 and the binding energy was calibrated using the C 1s peak at 284.6 eV.

### 2.3. Electrochemical measurements

Electrochemical measurements were performed in a three-electrode electrochemical cell using a Zahner ZENNIUM E electrochemical workstation. The electrodes for the electrochemical measurements were fabricated as follows. Catalyst ink was prepared by mixing catalyst powder with milli-Q water solution (1 ml for 10 mg of electrocatalyst). In total, 5% Nafion dispersion (DuPont) was added (50 mg solid Nafion for 100 mg of catalyst) to the catalyst slurry. Catalyst coating on glassy carbon electrode with 0.196 cm^2^ active area was fabricated by drop-casting the catalyst ink on with a micropipette. The catalyst loading was 0.4 mg catalyst cm^−2^. The electrode was then dried under vacuum at room temperature. The electrolytes for electrochemical measurements were prepared with perchloric acid (Aldrich) and Milli-Q water (Millipore). Pt foil (purity 99.999%) was purchased from Aldrich. All the electrochemical measurements were performed in 0.1 M HClO_4_ (aq) electrolyte, which was deaerated with hydrogen gas before use. A platinum wire, and an Ag/AgCl reference electrode (3 M KCl) were used as the counter electrode and reference electrode, respectively. All potentials, E, are quoted with respect to reversible hydrogen electrode (RHE). The electrocatalysis was studied using linear sweeping voltage in the range of +0.2 V to −0.5 V (versus RHE). Electrochemical impedance spectroscopy (EIS) was performed in potentiostatic mode at an applied overpotential of 100 mV with frequency from 10 mHz to 0.1 MHz and amplitude of 5 mV. The accelerated deterioration test of electrocatalysts was examined by applying cyclic voltammetry in the potential range from +0.1 to −0.5 V versus RHE for 1000 cycles.

## 3. Results and discussion

**Scheme 1.**
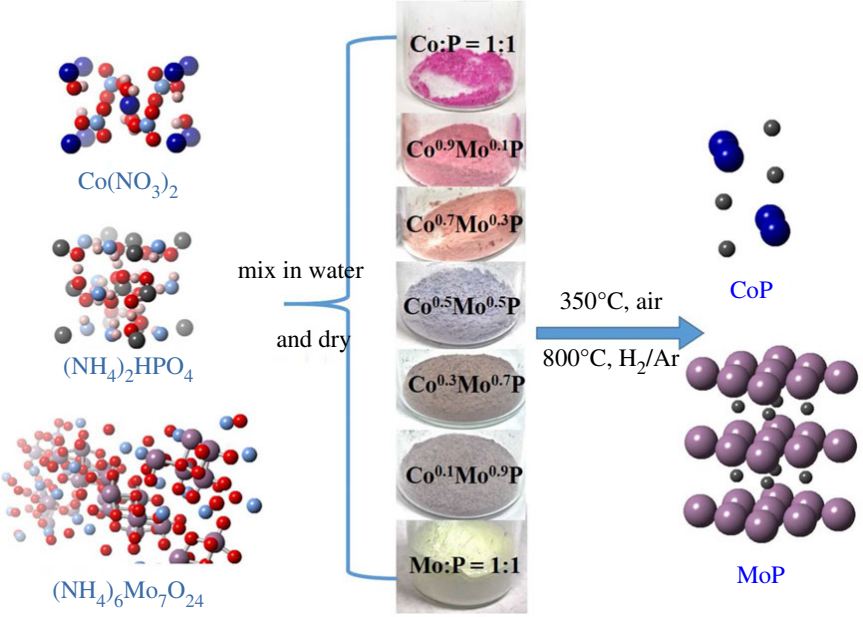
Schematic depictions of the synthesis of Co^φ^Mo^τ^P through treating a mixture of metal precursors and ammonium hydrogen phosphate with a two-steps solid-state process. The difference in colour of these precursor mixtures with different Co : Mo : P ratios is shown as the inset.

The goal of this investigation is combining Mo with Co in order to tune the properties of their phosphides. Early attempts found enhancement of the HER activity on MoP by the doping of small amounts of Co onto its hexagonal structure [20]. The synthesis was reported to produce hexagonal MoP, while the state of cobalt was not known. In this study, a set of catalysts was prepared with different cobalt-to-all-metal atomic ratios, *φ* (*φ* = [Co]/[Co + Mo], *τ* = [Mo]/[Co + Mo]). Co^φ^Mo^τ^P (e.g. Co^0.1^Mo^0.9^P) is defined as the sample code of the obtained catalysts; it does not represent any crystalline phase. The Co^φ^Mo^τ^P catalysts investigated were Co^0.1^Mo^0.9^P, Co^0.3^Mo^0.7^P, Co^0.5^Mo^0.5^P, Co^0.7^Mo^0.3^P and Co^0.9^Mo^0.1^P as shown in scheme 1.

**Figure 1.**
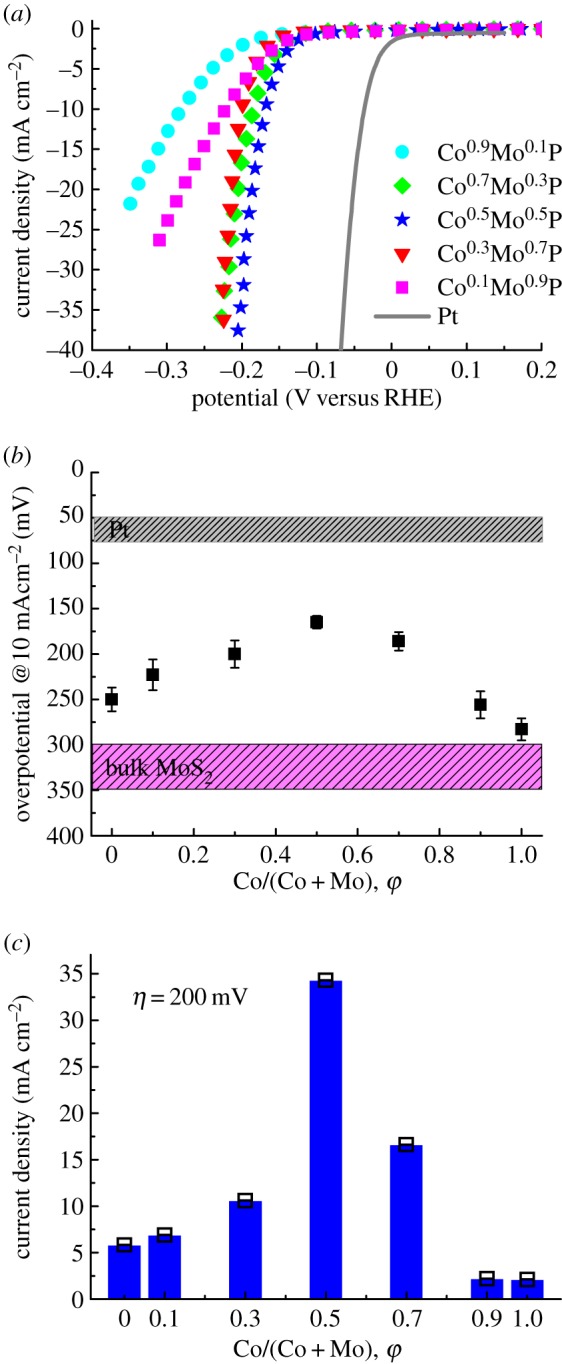
(*a*) Polarization curves of the Co^φ^Mo^τ^P catalysts annealed at 800°C and a Pt foil in hydrogen-purged 0.1 M HClO_4_. (*b*) Activity volcano for the HER showing the overpotentials from (*a*) at current density of 10 mA cm^−2^ as a function of cobalt content *φ*. The data of Pt and MoS_2_ from the literature are also included. (*c*) The dependence of current density at an overpotential of 200 mV from (*a*) on the cobalt content.

We investigated the HER activities of the Co^φ^Mo^τ^P catalysts, MoP (the same process for making Co^φ^Mo^τ^P) and CoP in 0.1 M HClO_4_ solution using a typical three-electrode electrochemical cell. The HER activity is compared using the overpotential (*η*) at 10 mA cm^−2^ of cathodic current density (*η*_10_) and the current density at *η* = 200 mV. For assessing the electrochemical analysis, in figure 1*a*, a Pt foil exhibits HER activity with *η*_10_ of 50 mV which is comparable to other studies. The polarization curve recorded with Co^φ^Mo^τ^P showed good activity for the HER. It is observed clearly in the electrochemical cell that the hydrogen bubbled more vigorously with increasing overpotentials. The *η*_10_ of the MoP and the CoP catalysts were read at 250 mV and 283 mV, respectively, which are similar to the overpotentials reported previously [29]. For Co^φ^Mo^τ^P catalysts, the polarization curves for the HER in figure 1*a* changed with the composition. In figure 1*b*, the overpotential, *η*_10_, is plotted versus the cobalt content, *φ*. The overpotential data of bulk MoS_2_ are collected from the literature [30]. It is observed that both bulk CoP and MoP show better HER activity than bulk MoS_2_. MoS_2_ is known as an edge-active material owing to its unique two-dimensional structure [31–34], while CoP and MoP are active in most facets. In figure 1*b*, it is shown that on adding Co to the MoP crystal (*φ* = 0 − 0.5), the *η*_10_ decreases (better activity) from 250 to 165 mV, and then it forms a volcano shape with maxima at *φ* = 0.5 as *φ* increases to 1.0. This result indicates that when mixing CoP and MoP, the HER activity is enhanced. The Co^0.5^Mo^0.5^P catalyst showed an *η*_10_ at 165 mV which rivals other bulk catalysts reported. Semimetallic MoP_2_ nanoparticles showed an *η*_10_ of 143 mV [35]. On CoS_2_ thin film, an *η*_10_ of 192 mV was observed by Faber *et al*. [36]. Very recently, Jiang *et al*. [28] demonstrated that Chevrel-phase bimetal sulfide NiMo_3_S_4_ possesses good activity with an *η*_10_ of 257 mV. Thus, the Co^0.5^Mo^0.5^P catalyst presented here is one of the top non-Pt HER catalysts in the bulk form. In figure 1*c*, the current density at *η* = 200 mV of the Co^φ^Mo^τ^P catalysts showed a volcano shape as a function of the Co content. The Co^0.5^Mo^0.5^P catalyst showed an activity of 34.3 mA cm^−2^ which is 5.9 and 16.3 times the activity of MoP and CoP catalysts, respectively.

**Figure 2.**
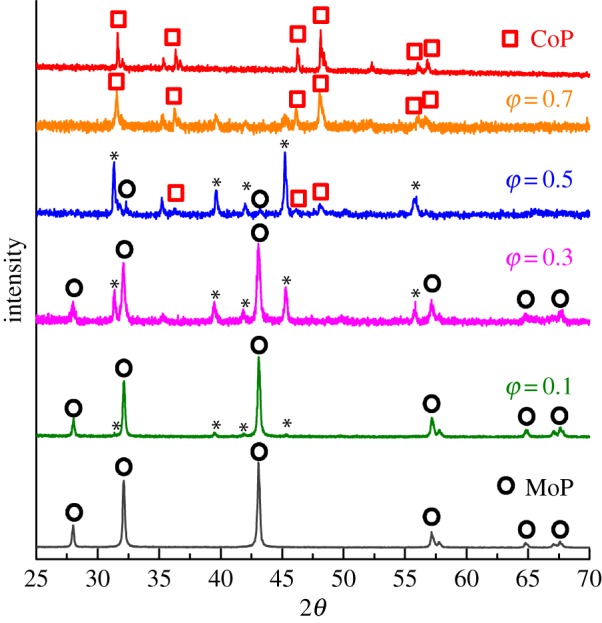
X-ray diffraction patterns corresponding to the Co^φ^Mo^τ^P catalysts annealed at 800°C as well as the single-phase MoP and CoP powder. The diffraction peaks are assigned to specific crystalline phases as follows. Black hollow circle, hexagonal MoP; red hollow square, orthorhombic CoP; black asterisk, hexagonal CoMoP_2_.

To understand how the composition affects the HER activity, XRD patterns were collected from these catalysts. The powder XRD studies in figure 2 evidence the presence of cobalt phosphide and molybdenum phosphide, and their relative peak intensities differ with the various ratios of Co/Mo. For *φ* from 0 to 0.3, the patterns suggest that the major crystal phase is WC-type MoP (ICSD No: 186 874), with *a* = 3.25 Å, *b* = 3.25 Å, *c* = 3.24 Å, and space group symmetry P6m2 (#187). For *φ* = 0.1, new diffraction peaks appeared at 2*θ* = 31.3, 39.7, 42.0, and 45.2° (marked as asterisk) with a very low intensity. The intensity of these peaks increases as *φ* increases and reaches maximum at *φ* = 0.5. These peaks were confirmed as hexagonal CoMoP_2_ structure (hereinafter *h*-CoMoP_2_ is used to represent this crystal phase in order to distinguish the difference from the hybrid composition Co^0.5^Mo^0.5^P) with *a* = 3.33 Å, *c* = 11.22 Å, and a space group P63/mmc (ICSD No: 624219, see the electronic supplementary material, figure S1). In addition to MoP and *h*-CoMoP_2_ phases at *φ* = 0.5, minor signals related to MnP-type orthorhombic CoP (ICSD No. 43249) also exist. For spectra with *φ* = 0.5–1.0, the intensity of CoP peaks increase, but *h*-CoMoP_2_ peaks decrease and MoP peaks disappear completely. The XRD pattern of the sample with *φ* = 0.9 is not shown here because it showed a similar pattern to CoP. The formation of ternary *h*-CoMoP_2_ phase was previously explained by the composition of trigonal-prismatic MoP_6_ and octahedral CoP_6_ prism to form intermetallic linear –Mo–Co–Mo– chains along the *c*-axis by Guérin & Sergent [37]. Interestingly, the *h*-CoMoP_2_ crystal possesses a similar trigonal-prismatic MoP_6_ prism to MoP crystal. X-ray photoelectron spectroscopy (XPS) was used to probe the surface electronic properties in the Co^0.5^Mo^0.5^P hybrid. The Co 2p spectrum (see the electronic supplementary material, figure S2*a*) showed two dominating peaks appeared at 777.0 and 791.8 eV corresponding to the Co 2p_3/2_ and Co 2p_1/2_ signals, respectively. The minor broad peak at 780 eV indicates the presence of surface oxidized Co species resulting from the contact with air. The Mo 3d spectrum, as shown in electronic supplementary material, figure S2*b*, showed a strong 3d_5/2_ peak at 227.8 eV and 3d_3/2_ peak at 231.1 eV. These peaks are assigned to zero valence state metallic Mo which indicates no or very little oxidation occurs on the Mo site. In the P 2p XPS spectrum shown in the electronic supplementary material, figure S2*c*, the strong peak at 129.4 eV is assigned to negative charged (metal-P^δ−^) phosphide. The minor peak at 133.4 eV is referred to the P–O species. The crystal field model describes that the strong P ligands split the Co 3d states in the octahedral CoP_6_ prism into high-spin t_2g_^5^e_g_^2^ ground state. Kibsgaard *et al*. [11] have shown that smaller differential hydrogen adsorption-free energies ΔG_H_ of the Co-bridge phosphorus site on CoP surface at a low coverage than that of the P site on MoP surface at a high coverage makes the CoP_6_ prism a high turnover-frequency towards the HER.

**Figure 3.**
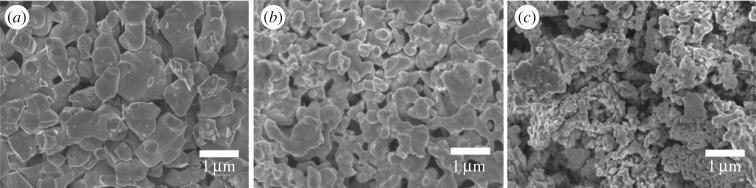
SEM images of (*a*) CoP, (*b*) Co^0.5^Mo^0.5^P and (*c*) MoP crystals taken at a magnification of 100 000*x*.

The reaction between the cobalt and/or molybdenum precursors with ammonium hydrogen phosphate under hydrogen environment at 800°C generated uniform phosphide microparticles. The SEM image in figure 3 shows that the CoP and the Co^0.5^Mo^0.5^P samples are composed of primary particles, but the MoP contains mostly secondary aggregated particles. These results indicate that the ratio of Co and Mo precursors affects the basic morphology of the catalysts. This can be ascribed to the complicated decomposition process regarding the reduction of MoO*_x_* in the high-temperature reduction reaction. The Co_0.5_Mo_0.5_P hybrid was characterized by transmission electron microscopy (TEM). The TEM image (see the electronic supplementary material, figure S3) showed interconnected structure composed of large particles (0.3–0.6 µm) which is in good agreement with the SEM result. The energy dispersive X-ray (EDX) spectra (as shown in electronic supplementary material, figure S4) collected on the TEM showed signals from Co, Mo and P elements. The Cu signals resulted from the Cu grid. The atomic percentages of Co, Mo and P elements obtained are 26.9, 24.2 and 48.9%, respectively, which answers to the composition of Co^0.5^Mo^0.5^P well.

**Figure 4:**
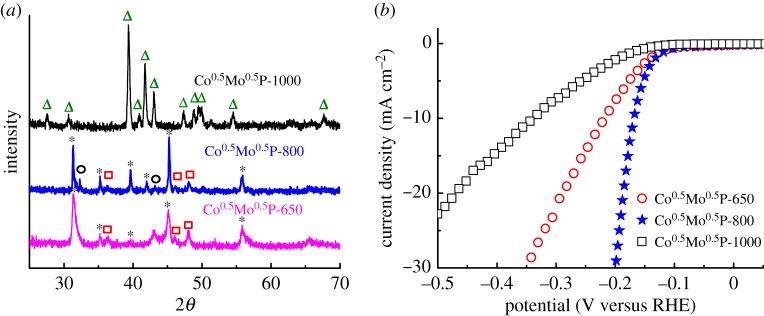
(*a*) X-ray diffraction patterns and (*b*) HER polarization curves of the Co^0.5^Mo^0.5^P samples annealed at 650°C, 800°C and 1000°C. The assignments of the diffraction peaks are included: black hollow circle, hexagonal MoP; red hollow square, orthorhombic CoP; black asterisk, hexagonal CoMoP_2_; green hollow triangle, orthorhombic CoMoP. The HER polarization curves were collected in hydrogen-purged 0.1 M HClO_4_ solution.

The effect of annealing temperature on the crystal structure and the HER activity of the Co^0.5^Mo^0.5^P catalyst are studied as shown in figure 4. The Co^0.5^Mo^0.5^P catalyst annealed at 650^o^C (Co^0.5^Mo^0.5^P-650, pink line in figure 4*a*) presents diffraction signals composed of *h*-CoMoP_2_ (black asterisk) and CoP (red hollow square) crystals. After annealed at 1000°C (Co^0.5^Mo^0.5^P-1000, black line in figure 4*a*), a new orthorhombic CoMoP with a Pnma space group (ICSD No. 2421) is formed (marked as green hollow triangle). The result indicates that increasing annealing temperature to 1000°C induced a phase transformation from *h*-CoMoP_2_ to CoMoP, and the excess phosphorus was evaporated. The polarization curves for the HER of Co^0.5^Mo^0.5^P-650, Co^0.5^Mo^0.5^P-800 and Co^0.5^Mo^0.5^P-1000 are compared in figure 4*b*. The HER activities of these catalysts are in series of Co^0.5^Mo^0.5^P-1000 < Co^0.5^Mo^0.5^P-650 < Co^0.5^Mo^0.5^P-800. The Co^0.5^Mo^0.5^P-1000 catalyst showed a poor HER activity with *η*_10_ of 338 mV, which indicates that CoMoP structure is less active than the P-rich *h*-CoMoP_2_. This can be explained by the higher phosphorus content in the *h*-CoMoP_2_ (28.6%) than that in CoMoP (16.7%). Callejas *et al*. [15] reported that CoP showed significantly lower overpotential than Co_2_P to produce the same current density. Xiao *et al*. [17] compared the HER activity between bulk MoP and Mo_3_P and concluded that MoP is more active than Mo_3_P. Thus, in this study, the *h*-CoMoP_2_ with higher P content showed better HER activity.

**Figure 5.**
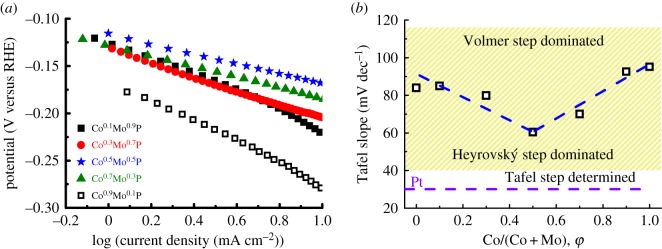
(*a*) Tafel plots of the Co^φ^Mo^τ^P catalysts derived from figure 1*a*. (*b*) The dependence of average Tafel slopes on the cobalt content, *φ*. The Tafel slope of a Pt electrode is also included.

The dependence of the gradient of logarithm of current density versus potential (so-called Tafel plot) corresponds to the reaction mechanism of the HER. The Tafel curves recorded on the Co^φ^Mo^τ^P catalysts (annealed at 800°C) as shown in figure 5*a* proceed in accordance with the classical two-electron-reaction model. The cathodic hydrogen evolution in acidic conditions involves either Volmer–Tafel (proton discharge followed by the recombination of two bound hydrogen) or Volmer–Heyrovský mechanism (proton discharge followed by electrochemical hydrogen desorption). Tafel slopes were obtained by linear fitting of the low current density region (−0.1 to +0.5 log[mA cm^−2^]). Figure 5*b* shows the dependence of the obtained Tafel slopes on the cobalt content, *φ*. For Pt foil, the Tafel slope of 30.1 mV dec^−1^ suggests a Volmer–Tafel mechanism that the recombination of two H_ads_ is rate-determining. The data in figure 5*b* showed decreasing Tafel slopes from 84.0 to 60.5 mV dec^−1^ for samples from *φ* = 0 to 0.5. These results suggest that at a small *φ* (*φ* = 0, high Tafel slope) hydrogen evolution occurs via a Volmer–Heyrovský mechanism in which slow adsorption of proton dominates the kinetics. For *φ* = 0.5, the small Tafel slope of 60.5 mV dec^−1^ indicates that the electrochemical desorption of hydrogen influences the reaction kinetics through the Volmer–Heyrovský mechanism much more than the case of MoP catalyst. Comparing the electrochemical properties of Co^0.5^Mo^0.5^P catalyst to bulk CoP and MoP catalysts, the small overpotential and the low Tafel slope of Co^0.5^Mo^0.5^P demonstrate that the presence of *h*-CoMoP_2_ crystal phase in the catalyst favours proton adsorption kinetics. To further ensure that *h*-CoMoP_2_ crystal phase enhances HER activity, we collected polarization curves from three 50/50 mixtures of CoP/MoP powder. The obtained polarization curves of the physical mixtures (see the electronic supplementary material, figure S5) showed HER activity ranging between pure CoP and MoP catalysts. This result indicates clearly that the physical mixture of CoP and MoP powder does not enhance the HER activity, while the *h*-CoMoP_2_ crystal phase does.

**Figure 6.**
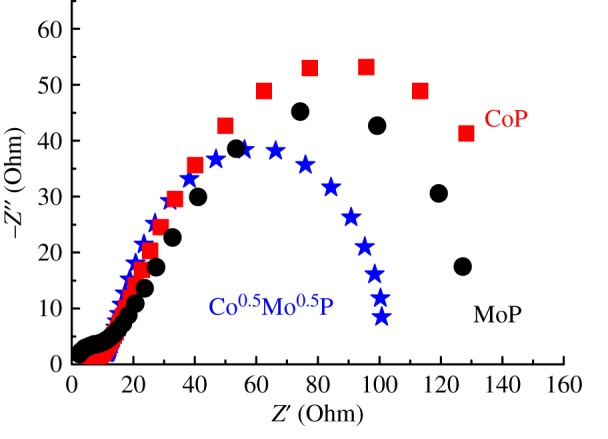
Comparison of the Nyquist plots of the Co^0.5^Mo^0.5^P, CoP and MoP catalysts at an overpotential of 100 mV in 0.1 M HClO_4_ solution.

We carried out EIS analyses on these catalysts to study the charge transfer in the catalytic turnover. The measurements were conducted at overvoltage of 100 mV. The Nyquist plots of the Co^0.5^Mo^0.5^P, CoP and MoP samples as shown in figure 6 revealed classical two time-constants characteristics that correspond to a combination of kinetic and diffusion response of the HER on rough electrode surfaces. This two time-constants equivalent circuit is included in the electronic supplementary material, figure S6. The series resistance, *R*_s_, is a sum of all ohmic resistances that the electron passed through from wires to the catalyst surface. The double-layer capacitance, *C*_d1_, in parallel with the charge transfer resistance, *R*_ct_, contributes to the charge-transfer process; the capacitance of the catalyst coating, *C*_d2_, in parallel with the resistance of ion conducting paths that develops in the porous surface, *R*_p_. The curves in figure 6 were fitted with the above two time-constant equivalent circuit. The fitting agrees well with the experimental results (see the electronic supplementary material, figure S7). These electrodes showed small series resistances between 2.5 and 4 Ω which indicates good adhesion of the powder on glassy carbon electrode. In figure 6, the pronounced semicircle at low frequencies (high Z’) returns estimates of the charge-transfer resistance, *R*_ct_. The *R*_ct_ of the Co^0.5^Mo^0.5^P catalyst (92.0 Ω) is found much lower than CoP (154.1 Ω) and MoP catalysts (107.5 Ω). As reported recently, the *R*_ct_ of bulk MoS_2_ was found to be 150 Ω at *η* = 150 mV [30]. MoS_2_/RGO has an Rct of 250 Ω at *η* = 120 mV [38]; Mo_2_C nanowires showed a low *R*_ct_ of 90 Ω at *η* = 150 mV [39]. Thus, the present Co^0.5^Mo^0.5^P catalyst is regarded as a catalyst with efficient charge transfer property. Efficient charge transfer kinetics reflects to the accelerating of the proton discharge step in the Volmer–Heyrovský mechanism and thus lowers its Tafel slope as aforementioned. The enhancement in *R*_ct_ can be ascribed to the modification of d-band structure owing to the formation of Co–P–Mo linkage.

**Figure 7.**
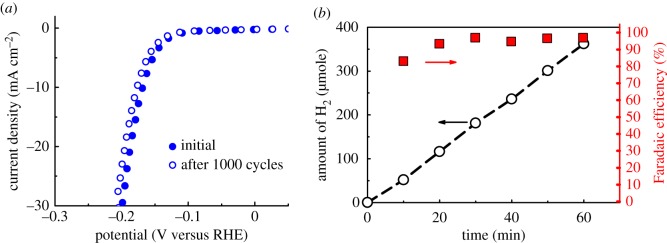
(*a*) HER polarization curves of Co^0.5^Mo^0.5^P electrode before and after potential sweeps (–0.5 ∼ +0.1 V versus RHE) for 1000 cycles in 0.1 M HClO_4_ solution (scan rate 2 mV s^−1^) (*b*). Time dependence of the quantity of hydrogen produced experimentally (dashed line) and the Faradaic efficiency of the Co^0.5^Mo^0.5^P catalyst at a fixed cathodic current density of 10 mA cm^−2^ in 0.1 M HClO_4_ solution 60 min.

To assess the stability of the Co^0.5^Mo^0.5^P electrodes, we performed the accelerated deterioration experiment by sweeping the applied potential from −0.5 to + 0.2 V versus RHE in 0.1 M HClO_4_ solution. After 1000 cycles on the Co^0.5^Mo^0.5^P electrode, only a slight shift (11 mV increase in *η*_10_) in the polarization curve is observed as shown in figure 7*a*, indicating a good stability of the Co^0.5^Mo^0.5^P catalyst in the operating conditions. Faradaic efficiency was determined by performing a controlled potential electrolysis experiment using an H-type cell. The amount of hydrogen gas produced was calculated from the volume of gas at a fixed cathodic current density of 10 mA cm^−2^ in 0.1 M HClO_4_ solution for 60 min. The experimentally determined hydrogen quantity was compared to the calculated amount based on the charge consumed as shown in figure 7*b*. The Faradaic efficiency reached 98% after 60 min of operation. The cathodic current density recorded for driving the HER at overpotential of 165 mV as a function of time is plotted in the electronic supplementary material, figure S8. The current density slightly decreased about 5% after 6 h of electrolysis. These results imply that the Co^0.5^Mo^0.5^P catalyst is a highly efficient and cathodically stable HER catalyst in the acidic environment.

## 4. Conclusion

We have studied the relationship between the composition of Co^φ^Mo^τ^P hybrids and their activity in the HER. The HER activity of the Co^φ^Mo^τ^P hybrids showed a volcano shape with a maxima at Co content of 0.5. The Co^0.5^Mo^0.5^P catalyst possesses a smaller overpotential (165 mV for driving 10 mA cm^−2^ of current density) when compared with CoP and MoP and is comparable to bulk non-precious catalysts reported. The hexagonal CoMoP_2_ phase was found to be responsible for the HER activity in the Co^0.5^Mo^0.5^P catalyst. The Tafel analysis showed a change in the dominating step in Volmer–Heyrovský mechanism. The low Tafel slope of the Co^0.5^Mo^0.5^P catalyst suggests that the electrochemical desorption of adsorbed hydrogen is the rate-determining step in the HER. The Co^0.5^Mo^0.5^P catalyst showed low charge transfer resistance, high stability and high Faradaic efficiency; all demonstrate that the Co^0.5^Mo^0.5^P catalyst is a potential, high-performance electrocatalyst for water electrolysis in acidic environments.

## Supplementary Material

Electronic Supplementary Materials including models and fitting of the electrochemical impedance spectroscopy, XRD, XPS, EDX spectra, and polarization curves.

## Data accessibility

Electronic supplementary material including models and fitting of the EIS, XRD, XPS, EDX spectra and polarization curves.
