# Supplementary material for: Enhanced hydrogen evolution reaction on hybrids of cobalt phosphide and molybdenum phosphide
Source: R Soc Open Sci. 2017 Mar 1;4(3):161016. doi: 10.1098/rsos.161016 (PMC5383849; doi:10.1098/rsos.161016)
Supplement: Electronic Supplementary Materials including models and fitting of the electrochemical impedance spectroscopy, XRD, XPS, EDX spectra, and polarization curves. [file rsos161016supp1.docx]

Electronic Supplementary Materials

**Enhanced Hydrogen Evolution Reaction on Hybrids of Cobalt Phosphide and Molybdenum Phosphide**

Si-Ling Fang,^1^ Tsu-Chin Chou,^1^ Satyanarayana Samireddi,^2,3^ Kuei-Hsien Chen,^1,2^ Li-Chyong Chen^1^ and Wei-Fu Chen^1^*

^1^ Center for Condensed Matter Sciences, National Taiwan University, Taipei 10617, Taiwan, Republic of China.

^2^ Institute of Atomic and Molecular Sciences, Academia Sinica, Taipei 10617, Taiwan, Republic of China.

^3^ Department of Chemistry, National Tsing Hua University, Hsinchu 30012, Taiwan, Republic of China.

*Corresponding author: Tel: +886–2–33665296; Fax: +886–2–2365–5404

E–mail: wfchen@ntu.edu.tw

**Figure S1** Comparison of XRD patterns between the Co^0.5^Mo^0.5^P hybrid and the ICSD *h*-CoMoP_2_ database (ICSD No. 624219).

**Figure S2** XPS spectra of the Co_0.5_Mo_0.5_P hybrid in the (a) Co 2P, (b) Mo 3d and (c) P 2P regions.


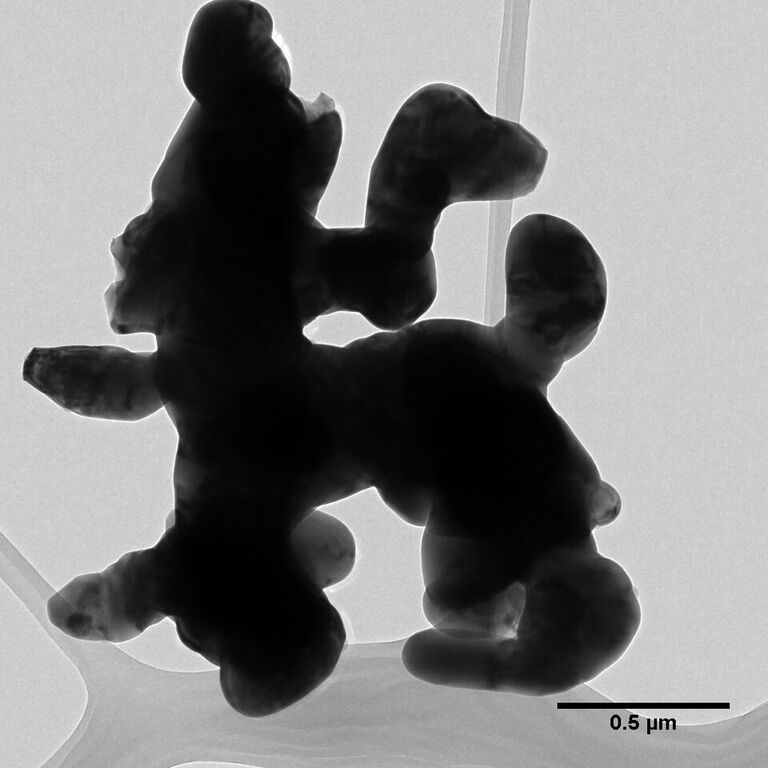


**Figure S3.** A TEM image of the Co_0.5_Mo_0.5_P hybrid.


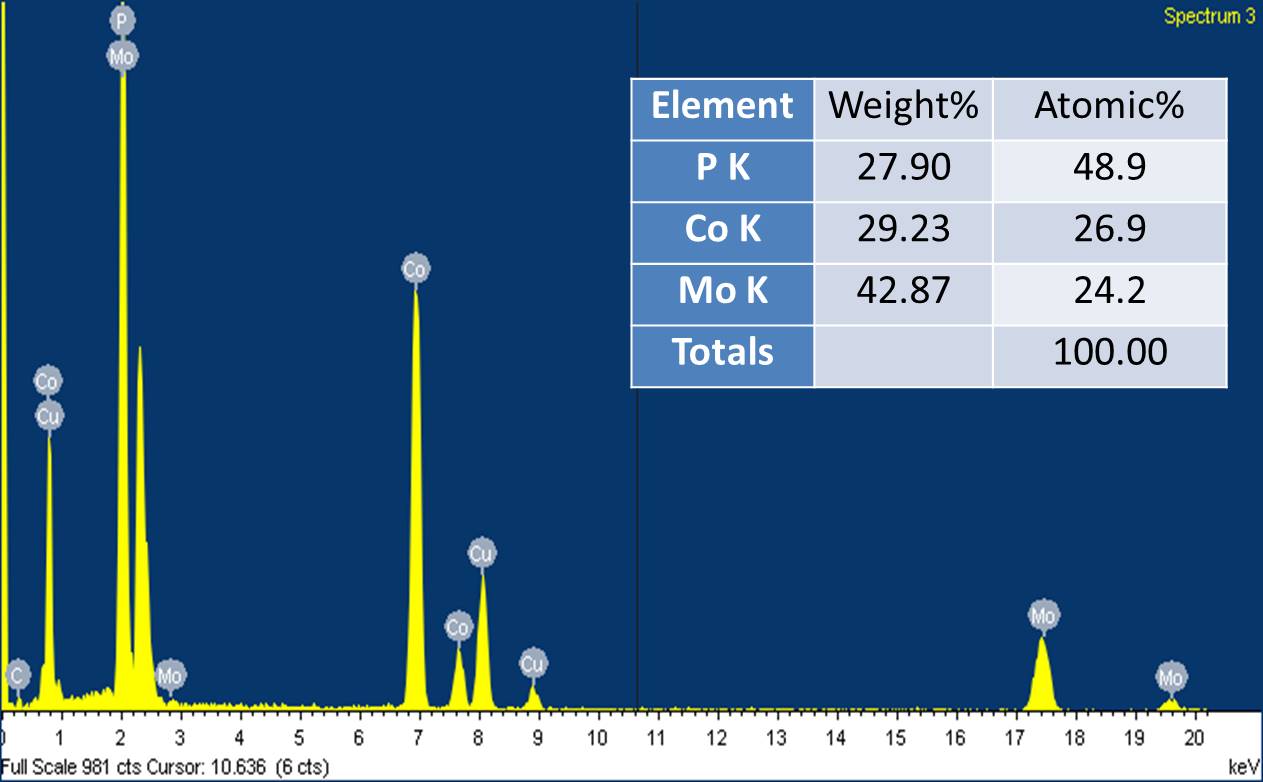


**Figure S4** EDX spectrum of the Co_0.5_Mo_0.5_P hybrid collected by a HRTEM, and the elementary compositions are shown in the inset.

**Figure S5** Comparison of polarization curves of the CoP, MoP and three physical mixtures with CoP:MoP = 1:1 (weight ratio) in hydrogen–purged 0.1 M HClO_4_.


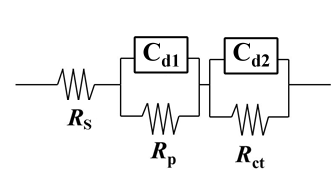


**Figure S6** The equivalent circuit model with two–time constant for fitting the electrochemical impedance spectroscopy under electrocatalytic condition in the hydrogen evolution reaction. *R*_s_, the series resistance; *R*_ct,_ the charge transfer resistance; *R*_p,_ resistance related to the surface roughness or porosity of the catalytic layer; *C*_d1_ and *C*_d2_, the double layer capacitances.

**Figure S7** Nyquist plots of experimental and simulated data for the (a) Co^0.5^Mo^0.5^P, (b) MoP (b) and (c) CoP annealed at 800^o^C simulated by the two-time constant model.

**Figure S8** Cathodic current density of the Co^0.5^Mo^0.5^P hybrid catalyst versus time at an overpotential of 165 mV for 6 hours.
